# Randomized Controlled Trial of Ultrasound-Guided Parasternal Intercostal Nerve Block and Transversus Thoracis Muscle Plane Block for Postoperative Analgesia of Cardiac Surgical Patients

**DOI:** 10.7759/cureus.72174

**Published:** 2024-10-23

**Authors:** Sachindra Yadav, Rajesh Raman, Rati Prabha, Dinesh Kaushal, Preeti Yadav, Sarvesh Kumar

**Affiliations:** 1 Department of Anesthesiology, Sukh Sagar Medical College and Hospital, Jabalpur, IND; 2 Department of Anesthesiology, King George's Medical University, Lucknow, IND; 3 Department of Anesthesiology, Netaji Subhash Chandra Bose Medical College, Jabalpur, IND; 4 Department of Cardiovascular and Thoracic Surgery, King George's Medical University, Lucknow, IND

**Keywords:** acute post-operative pain, cardiac surgery, fentanyl, intercostal nerves, regional anesthesia, ropivacaine

## Abstract

Background: Transversus thoracis muscle plane block (TTPB) and parasternal intercostal nerve block (PICNB) inhibit the anterior branches of intercostal nerves and potentially provide adequate analgesia after cardiac surgery. This study aimed to compare these two blocks for a reduction in postoperative opioid consumption after cardiac surgery.

Methods: This randomized, single-blind trial included 60 adult cardiac surgical patients divided into three groups to receive ultrasound-guided TTPB (group T), PICNB (group P), or no block (group C) before surgery. All patients received standard anesthesia with intravenous etomidate, fentanyl, midazolam, and vecuronium. Postoperative fentanyl consumption in the first 24 hours was the primary outcome variable. Secondary outcomes were pain fentanyl consumption in 48 hours, intensity, analgesia duration, heart rate, mean arterial pressure, and complications.

Results: The groups had similar baseline characteristics. The duration of analgesia was longer, while the intensity of pain and opioid consumption were statistically lower (p<0.01) in groups P and T compared to group C. The differences between groups P and T were not statistically significant. Fentanyl consumption in the first 24 hours was 284.00±37.61 µg, 293.00±35.11 µg, and 383.40±57.21 µg in groups P, T, and C, respectively. Other outcome variables were statistically similar among the groups.

Conclusion: Both TTPB and PICNB produce equivalent and satisfactory postoperative analgesia, reducing the postoperative fentanyl use in 24 hours for patients undergoing elective cardiac surgery.

## Introduction

Effective postoperative pain management in cardiac surgery is an important aspect of anesthesia for reducing patient morbidity and enhancing recovery. Thoracic epidural analgesia, the traditional method for perioperative analgesia, has been associated with serious complications such as hypotension and epidural hematoma, particularly in cardiac surgical patients on anticoagulation therapy [1]. This necessitates the search for alternative regional anesthesia techniques that provide adequate analgesia while minimizing the complications. Ultrasound-guided parasternal intercostal nerve block (PICNB) and transverse thoracis muscle plane block (TTPB) have emerged as promising approaches in regional anesthesia, targeting the anterior chest wall, a key area of postoperative pain in cardiac surgeries [2,3]. In PICNB, the drug is deposited between the pectoralis major (PM) and the external intercostal muscles, inhibiting the anterior intercostal nerves, which provide somatic sensation to the sternum and overlying skin [4,5]. In TTPB, the drug is deposited between the internal intercostal and transversus thoracis muscles to block the same nerves. Both blocks provide analgesia by blocking the anterior branches of T2-T6 intercostal nerves [5]. Both techniques have shown the potential to reduce opioid consumption and associated side effects. [3,6-10]. This randomized comparative trial aimed to compare postoperative opioid analgesic consumption after ultrasound-guided PICNB and TTPB in patients undergoing median sternotomy for cardiac surgery. The hypothesis of this study is that PICNB and TTPB reduce opioid consumption compared to standard care in patients undergoing elective cardiac surgery.

## Materials and methods

This prospective, single-blind, randomized controlled study was conducted in a tertiary care hospital. The trial was conducted after the ethics committee approval of King George's Medical University, Lucknow, India (approval number: 1219/Ethics/2020) and registration with the Clinical Trial Registry of India (CTRI/2021/05/033673). All patients gave informed and written consent, and the trial adhered to the ethical practice guidelines of The Declaration of Helsinki. The trial included adult (18-60 years) patients of either gender belonging to the American Society of Anesthesiologists (ASA) grades III or IV scheduled for cardiac surgery using median sternotomy. We excluded patients who were taking narcotic medications, undergoing redo-sternotomy, having a history of allergy to the study medications, obesity (BMI > 30 kg/m^2^), and refusing to give consent.

The recruited patients were assigned to one of the three groups to receive either bilateral PICNB (group P, n = 20), bilateral TTPB (group T, n = 20), or no nerve block (group C, n = 20) before surgery. The sixth author did patient recruitment. The second author performed randomization using a computer-generated random sequence in a 1:1:1 ratio. The allocation sequence was concealed using sealed opaque envelopes, which were opened by the consultant anesthesiologist after the patient's induction of anesthesia. The patients were blind to the study group allocation of the trial. A separate investigator, who was unaware of the intervention type, assessed postoperative pain and analgesic requirements. The postoperative ICU team managing the patients was also blinded to the study group of the patients.

After the arrival of the patients in the operating room, peripheral venous intravenous access was secured, and the patient was administered intravenous (IV) 1 µg/kg fentanyl and 0.04 mg/kg midazolam. Oxygen was given to the patients using a Hudson mask. An arterial cannula was inserted in the radial artery under local anesthesia for monitoring invasive blood pressure and blood sampling. Other monitoring included electrocardiogram, pulse oximetry, capnography, and temperature. Patients were induced with IV injections of fentanyl and etomidate. Muscle relaxation was achieved using IV vecuronium, and the patients were intubated using direct laryngoscopy. After induction of anesthesia, a femoral arterial line and central venous catheter were inserted in the internal jugular vein.

After this, the allocated block was administered to the patient by a consultant anesthetist. Bilateral PICNB (between pectoralis major and external intercostal muscle) and TTPB (between internal intercostal muscle and transversus thoracis muscle) were administered using the technique described by Toscano et al. [8]. A high-frequency (3-13 MHz) linear probe with a Mylab Seven (Esaote, Enrico Melen, Genova, Italy) ultrasound machine was used for all the blocks. 0.2% ropivacaine was used for all the blocks. Thirty ml of ropivacaine was given on each side for TTPB. Ten ml of ropivacaine was given for each of the six injections for PICNB. Surgery started after the administration of the blocks. Maintenance of anesthesia was achieved using a balanced anesthetic technique. During bypass, ventilation was stopped, and anesthesia was maintained using IV vecuronium, propofol, midazolam, and fentanyl. After the end of surgery, the patients were shifted to the postoperative cardiac ICU without extubation. The patients were given postoperative analgesia using IV paracetamol 1 gram bolus every six hours and 0.3-0.5 µg/kg/hour of IV fentanyl infusion till extubation. The pain intensity on the numeric rating scale (NRS) scale was assessed at eight, 16, 24, and 48 hours after surgery. However, if at any point after surgery, the patient requested analgesia for pain with NRS≥4, they were given an additional bolus of IV fentanyl (0.25-0.50 µg/kg). The time gap from the start of surgery to the first request for analgesia was noted as the duration of analgesia. After extubation and on the second postoperative day, the IV infusion of fentanyl was titrated between 0.3-0.5 µg/kg/hour to achieve a pan intensity <4 on NRS. If needed, an additional bolus of IV fentanyl (0.25-0.50 µg/kg) was given for analgesia.

The primary outcome variable of our study was the total amount of fentanyl used 24 hours after the end of surgery. Secondary outcome measures were duration of analgesia, total amount of fentanyl used in 48 hours, intensity of pain on the NRS scale, mean blood pressure, heart rate, and complications within 48 hours. The hemodynamic parameters were assessed at eight, 16, 24, and 48 hours after the end of surgery.

The current study had a power of 0.8 and a type I error of 0.05. A previous study had an analgesic consumption of 270±63 µg fentanyl with TTPB [11]. With the given standard deviation and to detect a difference in 60 µg in fentanyl consumption, a minimum of 18 patients were needed in each group. Twenty patients were recruited in each group to account for the data loss and patient exclusions. There were no changes to the study protocol after the start of the study.

Statistical analysis

For normally distributed continuous data, a one-way ANOVA was applied to compare means between the three groups. When the assumption of normality was violated, a Kruskal-Wallis test was used as a non-parametric alternative. Post hoc pairwise comparisons were done only for significant results. For nominal data, Fisher's exact test was used to compare proportions between groups. A p< 0.05 was considered statistically significant for all the statistical tests. Data were managed and analyzed in the IBM SPSS Statistics software for Windows, version 22.0 (IBM Corp., Armonk, NY). Data are presented as mean with SD or number with percentages.

## Results

**Figure 1:**
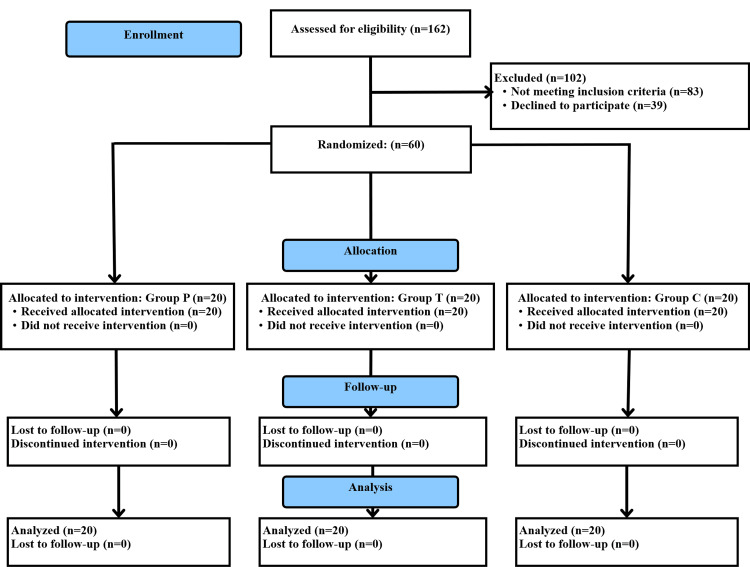
Flow of the participants in the trial group T: transversus thoracis muscle plane block group; group P: parasternal intercostal nerve block group; C: no block

**Figure 2:**
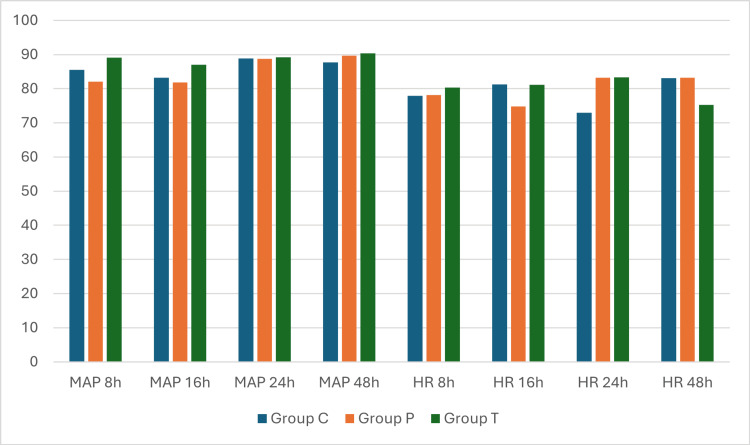
Comparison of hemodynamic variables MAP: mean arterial pressure in mmHg; HR: heart rate in beats per minute; h: hours; group T: transversus thoracis muscle plane block group; group P: parasternal intercostal nerve block group; C: no block

**Figure 3:**
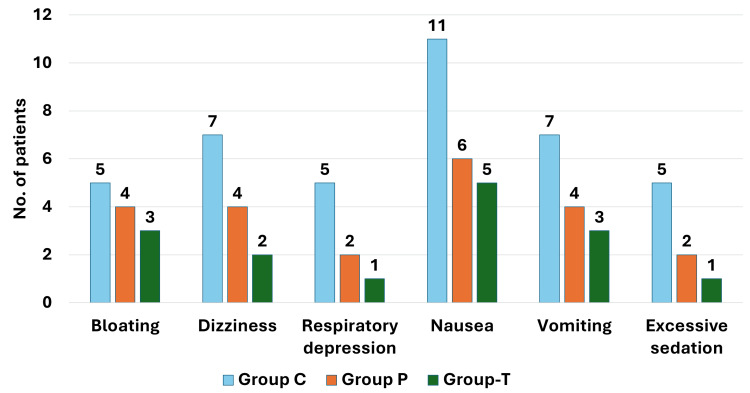
Comparison of complications group T: transversus thoracis muscle plane block group; group P: parasternal intercostal nerve block group; C: no block

The flow of participants in the study is shown in Figure 1. All the recruited patients were included in the analysis. The baseline and demographic characteristics of the patients were statistically comparable and are shown in Table 1. Duration of analgesia, postoperative fentanyl consumption, and pain intensity are shown in Table 2. The fentanyl use in the first 24 postoperative hours was 383.40±57.21, 284.00±37.61, and 293.00±35.11 µg in groups C, P, and T, respectively, with p<0.01. The duration of analgesia was 606.00±205.00, 1377.00±198.97, and 1335.00±231.87 minutes in groups C, P, and T, respectively, with p<0.01. The post hoc comparison of these outcomes is shown in Table 3. Postoperative pain at all the observed time points, duration of analgesia, and fentanyl consumption in 24 and 48 hours were higher in group C than in both groups P and T. No significant difference was found between groups P and T for fentanyl consumption in 24 and 48 hours, duration of analgesia, and postoperative pain scores. Postoperative hemodynamic variables (Figure 2) and complications (Figure 3) were comparable between the three groups. The complications were minor and self-limited. 

**Table 1:** Baseline and demographic characteristics of the participants BMI: body mass index; ASA: American Society of Anesthesiologists physical status; M: male; F: female; group T: transversus thoracis muscle plane block group; group P: parasternal intercostal nerve block group; C: no block

Parameter	Group C (n=20)	Group P (n=20)	Group T (n=20)	p-value
Age (years)	42.45±8.41	41.10±10.65	44.40±9.30	0.55
Height (cm)	152.90±8.41	151.90±4.67	151.95±5.85	0.86
Weight (Kg)	51.80±8.16	48.60±7.23	49.80±7.24	0.41
BMI (Kg/m^2^)	22.05±1.79	21.03±2.73	21.54±2.62	0.42
Gender (M/F)	8(40.0%)/12(60.0%)	9(45.0%)/11(55.0%)	11(55.0%)/9(45.0%)	0.64
ASA (III/IV)	15(75.0%)/5(25.0%)	9(45.0%)/11(55.0%)	10(50.0%)/10(50.0%)	0.14
Duration of surgery (minutes)	202.50±22.20	195.90±20.29	202.65±19.48	0.50
Intraoperative fentanyl (µg)	307.00±63.34	303.50±57.79	285.50±60.30	0.49
Type of surgery				0.93
Coronary artery bypass	5(25.0%)	4(20.0%)	3(15.0%)	
Mitral valve replacement	7(35.0%)	6(30.0%)	4(20.0%)	
Aortic valve replacement	4(20.0%)	4(20.0%)	5(25.0%)	
Atrial septal defect closure	2(10.0%)	2(10.0%)	4(20.0%)	
Ventricular septal repair	2(10.0%)	4(20.0%)	4(20.0%)	

**Table 2:** Comparison of the outcomes h: hours; pain is on the numeric rating scale; *: statistically significant; group T: transversus thoracis muscle plane block group; group P: parasternal intercostal nerve block group; C: no block

Parameter	Group C (n=20)	Group P (n=20)	Group T (n=20)	p-value
Fentanyl use in 24h (µg)	383.40±57.21	284.00±37.61	293.00±35.11	<0.01*
Fentanyl use in 48h (µg)	625.00±108.94	466.00±79.17	480.50±66.69	<0.01*
Duration of analgesia (minutes)	606.00±205.00	1377.00±198.97	1335.00±231.87	<0.01*
Pain 8h	4.95±1.10	3.95±1.00	3.75±0.97	<0.01*
Pain 16h	5.25±1.25	4.10±0.91	4.10±0.85	<0.01*
Pain 24h	5.95±1.05	4.15±1.14	3.90±0.97	<0.01*
Pain 48h	5.35±1.27	4.15±1.42	4.40±.94	0.01*

**Table 3:** Intergroup comparison h: hours; *: statistically significant; group T: transversus thoracis muscle plane block group; group P: parasternal intercostal nerve block group; C: no block

Groups	Group C vs. Group P	Group C vs. Group T	Group P vs. Group T
Duration of analgesia	<0.01*	<0.01*	0.81
Fentanyl use 24h	<0.01*	<0.01*	0.80
Fentanyl use 48h	<0.01*	<0.01*	0.86
Pain 8h	0.01*	<0.01*	0.811
Pain 16h	<0.01*	<0.01*	1.00
Pain 24h	<0.01*	<0.01*	0.74
Pain 48h	<0.01*	0.04*	0.80

## Discussion

In the current trial, we compared the TTPB and PICNB for pain relief after elective cardiac surgery. We observed that compared to the control, both the blocks resulted in lower consumption of opioid analgesics, lower pain scores, and a longer duration of analgesia. There was no difference in the clinical characteristics of the two blocks.

Ultrasound-guided truncal nerve blocks provide anesthesiologists with alternatives to central neuraxial blocks [12-16]. Despite providing a narrower width of analgesia, these blocks provide adequate analgesia for various surgeries of the chest and abdomen [13,16,17]. In addition, they have a lower risk of significant side effects, especially for patients receiving anticoagulants. As cardiac surgical procedures routinely use a high dose of heparin, the risk of bleeding associated with epidural analgesia is high. This makes the truncal nerve blocks an attractive option for postoperative analgesia.

Cardiac surgical procedures frequently require a midline sternotomy for access to the cardiac structures. This results in severe postoperative pain, which, if not managed well, can result in adverse perioperative outcomes and patient dissatisfaction [18]. The sensory nerve supply of the sternum and its overlying skin is primarily provided by the intercostal nerves [19]. These nerves, specifically the anterior cutaneous branches, pierce through the muscles and connective tissue near the sternum to innervate the skin overlying the sternum and the central part of the chest. Additionally, these nerves contribute to the sensation of the skin and subcutaneous tissue on the anterior thorax. The intercostal nerves are the anterior rami of the thoracic spinal nerves (T1-T11), which supply the muscles, skin, and parietal pleura of the thoracic wall. These nerves run in the intercostal spaces between the ribs, specifically along the costal groove located on the inferior edge of each rib [4]. Each intercostal nerve emerges from the spinal cord, travels between the internal and innermost intercostal muscles, and branches into lateral and anterior cutaneous branches to supply the overlying skin. In the anterior part of the chest wall, the anterior intercostal nerve lies between the transversus thoracis muscle and intercostal muscles. These nerves penetrate the intercostal muscles and pectoralis major muscle just lateral to the sternum and branch off into medial and lateral branches [5]. Anterior branches of the intercostal nerves can be blocked using local anesthetics just lateral to the sternum using either PICNB or TTPB. A PICNB blocks anterior intercostal nerves at a more superficial level between the intercostal nerves and the pectoralis major muscle. A TTPB blocks them between the transversus thoracis muscle and intercostal muscles. However, the internal mammary artery also runs between these two muscles at this level and can get damaged, leading to bleeding and hampering its harvesting for coronary artery bypass surgery [8]. As the drug is injected in a plane between the transversus thoracis and intercostal muscles, TTPB requires only one injection. Whereas, PICNB requires multiple injections to cover T2-T6 dermatomes effectively [7,8].

Both TTPB and PICNB for postoperative analgesia after cardiac surgery have been studied in a few trials. Toscano et al. compared TTPB and PICNB for postoperative analgesia on NRS as the primary outcome after adult cardiac surgery [8]. It was observed that the pain intensity on NRS was statistically similar to placebo in patients receiving the two nerve blocks. However, the control group had significantly higher morphine consumption at 24 and 48 hours postoperatively. In another study by Mansour et al., the two blocks were compared for postoperative analgesia in patients undergoing open cardiac surgery [7]. The total dose of morphine in the first 24 hours after surgery, the primary outcome variable, was significantly lower in the PICNB group than in TTPB. Postoperative pain on the Visual Analog Scale (VAS) was significantly higher in the TTPB group only at 18 hours. Complications were similar between the groups.

It was observed in our study that, compared to the control group, the intensity of pain on NRS was significantly lower in the TTPB and PICNB groups. As the major nerve supply to the sternum and overlying structures, the anterior intercostal nerves were effectively blocked by the two blocks, and the intensity of pain was significantly lower in our study. This finding contrasts with the observations of Toscano et al., who found no difference in pain scores than the control [8]. However, in their study, the consumption of morphine was higher in the control group, which may have led to similar pain scores to the patients receiving the nerve blocks.

The duration of analgesia was significantly longer in the patients receiving the nerve blocks in our study. Similar findings have been observed in previous studies comparing these blocks with placebo [2,3,6,15,19]. In a study comparing these two blocks, it was found that PICNB had a longer duration of analgesia than TTPB [7]. This contrasts with our findings. However, in their study, the authors used a higher volume of 0.25% bupivacaine (80 ml vs. 40 ml) for PICNB. This could have resulted in a longer duration of analgesia in the PICNB group.

In our study, opioid (fentanyl) consumption was significantly lower in patients receiving the blocks. Similar opioid-sparing effects were observed in a previous study [8]. However, in another study comparing the PICNB and TTPB, patients receiving PICNB had a lower morphine consumption [7]. This probably was a result of the use of a higher volume of 0.25% bupivacaine (80 ml vs. 40 ml) for PICNB in their trial.

The complications were similar between the three groups in our study, which may imply that the blocks are safer with few complications. Previous trials have not reported any increase in complications in patients receiving these two nerve blocks [2,3,6-8,15,19]. However, studies adequately powered to detect a meaningful difference are unavailable.

The study's limitations include its single-blind and single-center design. We also excluded obese patients from the study, limiting the generalizability of the trial findings to obese patients. 

## Conclusions

We observed that PICNB and TTPB provide adequate analgesia and reduce the dose of opioids for postoperative patients after cardiac surgery. The findings align with existing literature, demonstrating that both blocks are effective for pain management in cardiac surgery. We conclude that both blocks are suitable for providing postoperative analgesia in adult patients by reducing opioid consumption in the first 48 hours. However, larger studies are required to confirm the findings of this study and recommend these nerve blocks in routine practice for postoperative analgesia after cardiac surgery.
